# Insomnia and Neurocognitive Functioning in Adult Survivors of Childhood Cancer

**DOI:** 10.1093/jncics/pkaa008

**Published:** 2020-02-19

**Authors:** Ingrid Tonning Olsson, Margaret M Lubas, Chenghong Li, Belinda N Mandrell, Pia Banerjee, Carrie R Howell, Kirsten K Ness, Deokumar Srivastava, Leslie L Robison, Melissa M Hudson, Kevin R Krull, Tara M Brinkman

**Affiliations:** p1 Department of Epidemiology and Cancer Control, St. Jude Children’s Research Hospital, Memphis, TN, USA; p2 Department of Biostatistics, St. Jude Children’s Research Hospital, Memphis, TN, USA; p3 Department of Pediatric Medicine, St. Jude Children’s Research Hospital, Memphis, TN, USA; p4 Department of Oncology, St. Jude Children’s Research Hospital, Memphis, TN, USA; p5 Department of Psychology, St. Jude Children’s Research Hospital, Memphis, TN, USA

## Abstract

**Background:**

In noncancer populations, insomnia is known to affect neurocognitive processes. Although the prevalence of insomnia appears to be elevated in survivors of childhood cancer, relatively little is known about its association with neurocognitive performance in this at-risk population.

**Methods:**

A total of 911 survivors (51.9% female; mean [SD] age, 34 [9.0] years; time since diagnosis, 26 [9.1] years) completed direct assessments of attention, memory, processing speed, and executive functioning and self-reported symptoms of sleep (Pittsburgh Sleep Quality Index), fatigue (Functional Assessment of Chronic Illness Therapy-Fatigue), and daytime sleepiness (Epworth Sleepiness Scale). Sex-stratified general linear models were used to examine associations between insomnia and neurocognitive performance, with adjustment for treatment exposures and chronic health conditions. All statistical tests were two-sided.

**Results:**

Insomnia was reported by 22.1% of females and 12.3% of males (*P* < .001). After adjustment for neurotoxic treatment exposures, insomnia (vs healthy sleepers with no daytime fatigue or sleepiness) was associated with worse neurocognitive performance in the domains of verbal reasoning, memory, attention, executive function, and processing speed (verbal reasoning: males β = −0.34, *P* = .04, females β = −0.57, *P* < .001; long-term memory: males β = −0.60, *P* < .001, females β = −0.36, *P* = .02; sustained attention: males β = −0.85, *P* < .001, females β = −0.42, *P* = .006; cognitive flexibility: males β = −0.70, *P *=* *.002, females β = −0.40, *P* = .02). Self-reported sleep disturbance without daytime fatigue or sleepiness or daytime fatigue or sleepiness alone were not consistently associated with poorer neurocognitive performance.

**Conclusions:**

Insomnia was highly prevalent and contributed to the neurocognitive burden experienced by adult survivors of childhood cancer. Treatment of insomnia may improve neurocognitive problems in survivors.

Over 80% of children diagnosed with a malignancy will survive 5 years or more, contributing to a growing population of adult survivors of childhood cancer (1). However, 95% of these survivors will develop physical, neurocognitive, and/or psychosocial impairments by 45 years of age (2–4). Neurocognitive impairment, including deficits in attention, memory, and executive functioning, is highly prevalent among adult survivors of childhood cancer, particularly survivors who were exposed to neurotoxic treatments (eg, cranial radiation therapy, intrathecal and/or high-dose methotrexate) (5,6). Among survivors of adult-onset cancer, sleep disturbances are common (7); however, considerably less is known about sleep in adult survivors of childhood cancer. The Childhood Cancer Survivor Study reported that survivors experience poorer sleep quality compared with siblings, with 16.7–17.4% reporting poor sleep quality and 13.8–19.0% reporting daytime fatigue (8,9). In a sample of adult survivors of childhood cancer, excluding central nervous system tumors (n = 122), 28% reported clinically significant symptoms of insomnia defined as self-reported sleep efficiency less than 85% (10). Although initial research suggests pervasive self-reported sleep complaints among adult survivors of childhood cancer (8–10), the potential functional consequences of sleep disturbances in this population are poorly understood.

In noncancer populations, insomnia has been associated with impaired neurocognitive performance (11), with deficits reported in working memory (12,13), shifting or switching attention (13,14), vigilance (15), response inhibition (16), and memory (17). In addition, data from functional neuroimaging studies suggest altered activation and neural network connectivity in individuals with insomnia (12,18,19). Among survivors of adult-onset cancers, survivors with insomnia are 16 times more likely to report memory problems than survivors without insomnia (20). However, to our knowledge, only two reports have examined associations between sleep and neurocognition in survivors of childhood cancer. In a sample of adolescent survivors of childhood leukemia, poor sleep and fatigue were associated with worse performance on multiple neurocognitive measures in female survivors, and less robust associations were observed in male survivors (21). A report from the Childhood Cancer Survivor Study noted the association of poor sleep quality and increased risk of problems with attention and memory (8). However, this study relied on self-report of cognitive problems, the validity of which may be affected by the presence of cognitive impairment.

Because neurocognitive impairments in childhood cancer survivors may be exacerbated in the context of poor sleep (8,21), further research is needed among survivors to examine these associations independent of established risk factors, including neurotoxic therapies and chronic health conditions (22,23). Moreover, research is needed to identify whether insomnia specifically or a more general sleep disturbance is associated with neurocognitive impairment. For example, determining whether sleep disturbance, the daytime consequence, or the combination of both (eg, insomnia) is associated with decreased neurocognitive functioning could help inform future intervention development. Thus, the aim of the current study was to examine associations between insomnia, sleep disturbance, daytime sleepiness and/or fatigue, and performance-based neurocognitive outcomes in adult survivors of childhood cancer.

## Methods

### Study Participants

This study used the St. Jude Lifetime Cohort Study (SJLIFE), a retrospectively identified dynamic cohort with prospective medical follow-up. The cohort was initiated in 2007 and originally included survivors of childhood cancer who were 18 years of age and older, and at least 10 years postdiagnosis of a pediatric cancer treated at St. Jude Children’s Research Hospital. A further detailed description of the cohort and methodology was published previously (24–26). For our study, survivors previously enrolled in SJLIFE were recruited for an intervention study focused on sleep and neurocognition. We identified and screened 3348 potentially eligible SJLIFE participants (dates of diagnosis: 1964–2005; median years from diagnosis, 24.6, range = 10.4–50.8). Among these, 562 refused participation in the intervention and 1875 did not meet study-specific inclusion or exclusion criteria (see Supplementary Table 1 [available online] for a detailed list of intervention inclusion/exclusion criteria). This resulted in 911 participants who completed in-person baseline evaluations to confirm eligibility for the intervention study. This study reports on these 911 participants. All data were collected between February 2013 and October 2016. All participants provided written, informed consent, and the study was approved by the institutional review board at St. Jude Children’s Research Hospital.

### Neurocognitive Outcomes

Neurocognitive measures assessed verbal reasoning, memory, attention, processing speed, and executive functioning. The following tests were used: Wechsler Abbreviated Scale of Intelligence (vocabulary) (27); Conner’s Continuous Performance Test II (variability [sustained attention], omissions [inattention], detectability [selective attention] (28); Trail Making Test Part A [focused attention], Part B [cognitive flexibility]) (29); Wechsler Adult Intelligence Test-III (digit span forward [memory span], digit span backward [working memory], digit symbol coding [visuomotor processing speed], symbol search [cognitive processing speed]) (27); Grooved Pegboard Test (dominant hand [fine motor processing speed]) (30); California Verbal Learning Test-II (total [verbal learning], short-delay free recall [short-term memory], long-delay free recall [long-term memory]) (31); Controlled Oral Word Association Test (FAS [cognitive fluency]) (30). Scores from all measures were converted into age-adjusted Z scores (Mean = 0, [SD = 1.0]) and treated as continuous variables in the multivariable models.

### Insomnia, Sleep Disturbance, Fatigue, and Daytime Sleepiness

Insomnia, sleep disturbance, fatigue, and daytime sleepiness were measured with three different questionnaires: the Pittsburgh Sleep Quality Index (32), Functional Assessment of Chronic Illness Therapy-Fatigue (33,34), and Epworth Sleepiness Scale (35), respectively. Three questions from the Pittsburgh Sleep Quality Index were used to calculate sleep efficiency (the ratio of total sleep time to time in bed), usual bedtime, usual time getting up in the morning, and hours of sleep per night. The Functional Assessment of Chronic Therapy-Fatigue is a measure of physical and functional consequences associated with fatigue. The 13 items comprise a total score from 0 to 52, with lower scores indicating more fatigue. A cut-off score of less than 30 was used to identify clinically significant daytime fatigue (36). The Epworth Sleepiness Scale measures daytime sleepiness and likelihood of falling asleep during routine daily situations. The eight items on this measure generate a total score from 0 to 24, with higher scores indicating increased daytime sleepiness. A cut-off score greater than or equal to 10 was used to identify clinically significant daytime sleepiness (35). Insomnia was defined as a sleep efficiency less than 85% (32) and daytime impairment defined by daytime fatigue or sleepiness. This value corresponds to a sleep efficiency that would warrant clinical treatment for insomnia (10,37), and clinical definitions of insomnia require that the sleep disturbance is comorbid with a daytime consequence.

Using the above measures, survivors were categorized as 1) having insomnia (sleep efficiency <85% and daytime impairment); 2) having sleep disturbance (sleep efficiency <85% without daytime fatigue or sleepiness); 3) having daytime sleepiness or fatigue only; or 4) healthy sleepers (sleep efficiency >85% and no daytime sleepiness and/or fatigue).

### Covariates

Treatment exposures known to influence cognitive outcomes in survivors (38,39) were selected a priori: cumulative high dose of methotrexate (g/m^2^), cumulative dose of intrathecal methotrexate and/or cytarabine (mL), and cranial radiation dose (Gy). Measures of psychological distress, physical inactivity, amputation-adjusted body mass index, pain, and chronic health conditions were also included. The Brief Symptom Inventory 18 (40) was administered to assess symptoms of anxiety and depression. A T-score of 63 or greater on the individual subscales was considered to represent clinically significant anxiety or depression. Physical inactivity was defined as not meeting the Centers for Disease Control recommendation for physical activity (75 minutes of vigorous or 150 minutes of moderate activity per week). Weekly moderate and vigorous activities were converted into metabolic equivalents, and individuals who reported less than 450 metabolic equivalents per week were classified as inactive. Pain was defined as the presence of moderate to severe headaches (ie, migraines, severe headaches, repeated headaches) or other bodily pain (ie, prolonged pain in arms or legs, prolonged pain in back). Chronic health conditions were coded using a modification of the Common Terminology Criteria for Adverse Events (25) for cardiac-, respiratory-, and endocrine-related systems (Common Terminology Criteria for Adverse Events grades 0–1 vs 2–4). Beyond treatment exposures, covariates were included in analyses only if these data were collected within 3 months of the baseline sleep and neurocognitive measures.

### Statistical Analysis

Descriptive statistics were calculated for all exposures, outcomes, and covariates. Sex differences in insomnia symptoms and neurocognitive performance were assessed using the Student *t* test for continuous variables and χ^2^ for categorical variables. All tests were two-sided, and a *P* value of less than .05 was considered statistically significant. All analyses were stratified on sex because of previously observed sex differences in insomnia prevalence (41) and cognitive late effects to cancer treatment (42). Two sets of general linear multivariable models were used to examine associations between insomnia and neurocognitive outcomes. In the first set of models, associations between insomnia and neurocognitive outcomes were examined with adjustment for primary cancer treatment variables (high dose and intrathecal methotrexate or cytarabine, and cranial radiation therapy), age at diagnosis, and age at study. In the second set of models, associations between insomnia and neurocognitive outcomes were examined with adjustment for chronic health conditions, age at study, depression, anxiety, pain, and physical inactivity. All analyses were completed using SAS v9.4 and SPSS version 22 (43).

## Results

### Survivor Characteristics

Demographic and treatment characteristics are shown in Table 1. Survivors were an average (SD) of 34 (9.0) years of age, 26 (9.1) years from their initial cancer diagnosis, and 51.9% female. Of survivors, 41.4% were diagnosed with leukemia, 27.6% were treated with cranial radiation therapy, 31.6% received high-dose intravenous methotrexate, and 43.6% received intrathecal methotrexate and/or cytarabine.

**Table 1.** Demographic, treatment, and health characteristics of a sample of survivors from the SJLIFE* Cohort

	Total sample	Females	Males	
Variable	N (%)	N (%)	N (%)	*P* †
	n = 911	n = 473	n = 438	
Mean age at evaluation, y (SD)	34.29 (9.0)	34.25 (9.2)	34.33 (8.7)	.90
Mean age at diagnosis, y (SD)	8.76 (5.7)	8.50 (5.6)	9.04 (5.8)	.15
Mean time since diagnosis, y (SD)	25.51 (9.1)	25.74 (9.3)	25.26 (8.9)	.42
Race or ethnicity				.05
White, non-Hispanic	765 (84.0)	384 (81.2)	381 (87.0)	
Black	121 (13.3)	75 (15.9)	46 (10.5)	
Other	25 (2.7)	14 (3.0)	11 (2.5)	
Diagnosis				.35
Leukemia	377 (41.4)	188 (39.8)	189 (43.2)	
CNS tumor	70 (7.7)	31 (6.6)	39 (8.9)	
Non-CNS solid tumor	212 (23.3)	123 (26.0)	89 (20.3)	
Hodgkin lymphoma	118 (13.0)	62 (13.1)	56 (12.8)	
Non-Hodgkin lymphoma	56 (6.2)	28 (5.9)	28 (6.4)	
Ewing or osteosarcoma	63 (6.9)	35 (7.4)	28 (6.4)	
Other	15 (1.7)	6 (1.30)	9 (2.1)	
Radiation				
Cranial radiation				.06
≥20 Gy	138 (15.8)	60 (13.1)	78 (18.8)	
<20 Gy	103 (11.8)	58 (12.7)	45 (10.9)	
No cranial radiation	631 (72.4)	340 (74.2)	291 (70.3)	
Chest radiation	184 (21.1)	98 (21.4)	86 (20.7)	.79
Chemotherapy				
HD or IV methotrexate	288 (31.6)	142 (30.0)	146 (33.3)	.28
Intrathecal methotrexate or cytarabine	397 (43.6)	194 (41.0)	203 (46.4)	.10
Corticosteroids	478 (52.5)	243 (51.4)	235 (53.7)	.49
Anthracyclines	576 (63.2)	298 (63.0)	278 (63.5)	.88
Alkylating agents	538 (59.1)	279 (59.0)	259 (59.1)	.96
BMI, amputation adjusted, kg/m^2^	28.74 (7.1)	28.68 (7.7)	28.79 (6.4)	.81
Physically inactive‡‖	354 (44.9)	209 (51.7)	145 (37.7)	<.001
Psychological distress§‖				
BSI anxiety	50 (6.8)	28 (7.3)	22 (6.3)	.61
BSI depression	55 (7.5)	27 (7.0)	28 (8.0)	.60
Pain‖				<.001
Headache	173 (21.7)	129 (31.4)	44 (11.4)	
Other bodily pain	71 (8.9)	26 (6.3)	45 (11.7)	
Chronic conditions				
Cardiac conditions				.007
Grade <2	541 (59.4)	301 (63.6)	240 (54.8)	
Grade ≥2	370 (40.6)	172 (36.4)	198 (45.2)	
Endocrine conditions				.10
Grade <2	451 (49.5)	222 (46.9)	229 (52.3)	
Grade ≥2	460 (50.5)	251 (53.1)	209 (47.7)	
Respiratory conditions				.14
Grade <2	670 (73.6)	338 (71.5)	332 (75.8)	
Grade ≥2	241 (26.5)	135 (28.5)	106 (24.2)	

* BMI = body mass index; BSI = Brief Symptom Inventory; CDC = Centers for Disease Control; CNS = central nervous system; HD = high dose; IV = intravenous; MET = metabolic equivalent; SJLIFE = St. Jude Lifetime Cohort Study.

† Chi-square test; all tests were two-sided.

‡ Physically inactive was defined according to CDC criteria of 450 MET-min/wk.

§ Psychological distress defined as T score of at least 63.

‖ The following variables had greater than 10% missing: physically inactive (n = 122); anxiety and depression (n = 176); pain (n = 114).

### Insomnia and Daytime Sleepiness or Fatigue

Insomnia (ie, sleep efficiency <85% and daytime fatigue or sleepiness) was reported by 17.4% of survivors. Among females, 22.1% reported insomnia, 29.0% reported a sleep disturbance without daytime sleepiness or fatigue, and 14.5% reported daytime fatigue or sleepiness only. The corresponding figures for males were 12.3%, 26.6%, and 13.1% (*P* < .001; Table 2).

**Table 2.** Prevalence of insomnia, daytime sleepiness and fatigue*

Variable	Total sample†	Females	Males
	n = 833	n = 435	n = 398
	No. (%)	No. (%)	No. (%)
No sleep disturbance, no daytime impairment	341 (40.9)	150 (34.5)	191 (48.0)
Daytime fatigue or sleepiness w/o SE<85%	115 (13.8)	63 (14.5)	52 (13.1)
Sleep disturbance, SE <85%	232 (27.9)	126 (29.0)	106 (26.6)
Insomnia, SE <85%, with daytime impairment	145 (17.4)	96 (22.1)	49 (12.3)

* Difference between females and males: *P *<* *.001, chi-square test. SE = sleep efficiency; w/o = without.

† Seventy-two individuals were removed from analyses due to reporting a SE greater than 100% on the Pittsburgh Sleep Quality Index; six individuals had missing sleep data.

### Neurocognitive Performance

**Figure 1.**
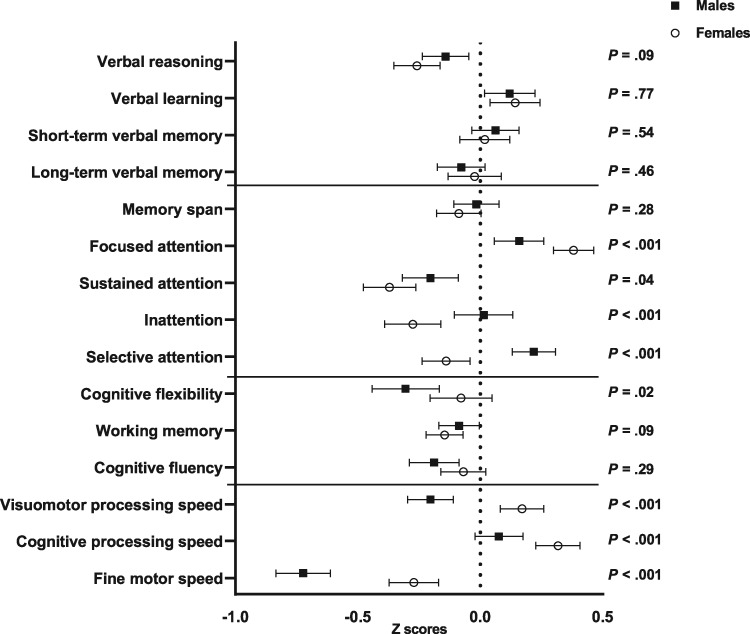
Neurocognitive performance by sex. Neurocognitive scores for males (n = 436) and females (n = 470), separately (missing neurocognitive data, n = 5). All scores are presented as Z scores (expected Mean = 0, [SD = 1]) with 95% confidence intervals. Higher scores represent better performance. *P* values represent statistical differences between males and females. All tests were two-sided.

Neurocognitive performance data are shown in Figure 1. Females performed better than males on one measure of attention (focused attention, *P *<* *.001), whereas males performed better than females on sustained attention (*P *=* *.04), inattention (*P* < .001), and selective attention (*P *<* *.001). In the domain of executive functioning, females performed better than males on cognitive flexibility (*P *=* *.02), and females performed better on all measures in the domain of processing speed (all *P* < .001). The prevalence of neurocognitive impairment by sex is shown in Supplementary Table 2 (available online).

### Multivariable Analyses

After adjustment for treatment exposures (Table 3), insomnia was associated with worse neurocognitive performance in males and/or females on 14 out of 15 measures. Sleep disturbance was associated with poorer neurocognitive performance across only four measures, and daytime sleepiness and/or fatigue was not associated with decreased neurocognitive performance across any measure. Among males, insomnia was associated with an approximately one-half SD worse performance on measures of verbal memory (verbal learning: β = −0.64, *P *<* *.001; short-term memory: β = −0.53, *P *=* *.002; long-term memory: β = −0.60, *P *<* *.001), a one-third to one-half SD worse performance on processing speed (visuomotor: β = −0.39, *P *=* *.02; cognitive processing: β = −0.40, *P *=* *.02; fine motor speed: β = −0.57, *P *=* *.002), a one-third to two-thirds SD worse performance on measures of executive function (cognitive flexibility: β = −0.70, *P* = .002; cognitive fluency: β = −0.36, *P *=* *.04; verbal reasoning: β = −0.34, *P* = .04), and a one-half to one full SD worse performance on measures of attention (focused: β = −0.54, *P *=* *.001; sustained: β = −0.85, *P *<* *.001; inattention: β = −1.09, *P *<* *.001). Among females, insomnia was associated with an approximately one-third to one-half SD worse performance on measures of verbal reasoning (β = −0.57, *P *<* *.001; memory span: β = −0.35, *P *=* *.009; inattention: β = −0.49, *P *=* *.001; sustained attention: β = −0.42, *P* = .006; cognitive flexibility: β = −0.40, *P = *.02; working memory: β = −0.30 *P *=* *.006; long-term memory: β = −0.36, *P* = .02; and processing speed [visuomotor: β = −0.45, *P *<* *.001; fine motor: β = −0.33, *P *=* *.02]). In multivariable models adjusted for chronic health conditions, physical inactivity, emotional distress, and pain, observed associations between insomnia and cognitive performance were similar, though somewhat attenuated (Table 4). Due to the comorbidity between emotional distress and insomnia, we conducted post hoc analyses removing anxiety and depression from our multivariable models (Supplementary Table 3, available online). In doing so, the association between insomnia and poorer neurocognitive performance strengthened and achieved statistical significance across five measures (verbal learning, memory span, cognitive flexibility, working memory, and cognitive processing speed) among females only.

**Table 3.** Associations between insomnia and neurocognitive performance with adjustment for neurotoxic treatment exposures

Outcome variable	Referent = no sleep disturbance, no daytime fatigue or sleepiness	Female*		Male†	
		β‡	*P*	β‡	*P* §
Verbal reasoning	Daytime sleepiness and/or fatigue	−0.04	.77	−0.08	.63
	Sleep disturbance	−0.26	.04	−0.17	.18
	Insomnia	−0.57	<.001	−0.34	.04
Verbal learning	Daytime sleepiness and/or fatigue	−0.01	.95	0.11	.52
	Sleep disturbance	−0.19	.17	0.01	.91
	Insomnia	−0.39	.009	−0.64	<.001
Short-term verbal memory	Daytime sleepiness and/or fatigue	0.00	.98	0.06	.70
	Sleep disturbance	−0.32	.02	−0.03	.84
	Insomnia	−0.35	.02	−0.53	.002
Long-term verbal memory	Daytime sleepiness and/or fatigue	0.11	.54	0.12	.46
	Sleep disturbance	−0.15	.30	0.02	.90
	Insomnia	−0.36	.02	−0.60	<.001
Memory span	Daytime sleepiness and/or fatigue	0.01	.96	0.02	.88
	Sleep disturbance	−0.27	.03	−0.00	.98
	Insomnia	−0.35	.009	−0.36	.03
Focused attention	Daytime sleepiness and/or fatigue	−0.06	.62	−0.11	.47
	Sleep disturbance	−0.04	.71	−0.03	.81
	Insomnia	−0.22	.06	−0.54	.001
Sustained attention	Daytime sleepiness and/or fatigue	−0.23	.18	0.29	.14
	Sleep disturbance	−0.19	.18	0.15	.31
	Insomnia	−0.42	.006	−0.85	<.001
Inattention	Daytime sleepiness and/or fatigue	−0.32	.07	0.03	.86
	Sleep disturbance	−0.17	.24	−0.03	.83
	Insomnia	−0.49	.001	−1.09	<.001
Selective attention	Daytime sleepiness and/or fatigue	−0.02	.90	0.26	.07
	Sleep disturbance	−0.08	.56	0.04	.74
	Insomnia	−0.12	.38	−0.15	.32
Cognitive flexibility	Daytime sleepiness and/or fatigue	−0.11	.56	0.13	.55
	Sleep disturbance	0.12	.46	0.06	.74
	Insomnia	−0.40	.02	−0.70	.002
Cognitive fluency	Daytime sleepiness and/or fatigue	0.10	.53	−0.09	.60
	Sleep disturbance	0.14	.25	−0.13	.34
	Insomnia	−0.22	.10	−0.36	.04
Working memory	Daytime sleepiness and/or fatigue	−0.03	.84	0.01	.95
	Sleep disturbance	−0.03	.77	−0.28	.01
	Insomnia	−0.30	.006	−0.26	.08
Visuomotor processing speed	Daytime sleepiness and/or fatigue	−0.19	.18	0.02	.91
	Sleep disturbance	−0.07	.52	−0.02	.87
	Insomnia	−0.45	<.001	−0.39	.02
Cognitive processing speed	Daytime sleepiness and/or fatigue	−0.06	.67	0.05	.74
	Sleep disturbance	0.09	.43	−0.10	.43
	Insomnia	−0.28	.03	−0.40	.02
Fine motor speed	Daytime sleepiness and/or fatigue	−0.19	.22	0.15	.41
	Sleep disturbance	−0.19	.13	−0.06	.68
	Insomnia	−0.33	.02	−0.57	.002

* Female models are based on a sample of n = 435 survivors with complete sleep and neurocognitive data; additional missing data due to covariates n = 22.

† Male models are based on a sample of n = 398 survivors with complete sleep and neurocognitive data; additional missing data due to covariates n = 27.

‡ Standardized β of neurocognitive measures are based on Z scores (Mean = 0, [SD = 1]). Separate models for each neurocognitive outcome, adjusted for age at diagnosis and age at survey (years), cumulative high dose methotrexate (g/m^2^), cumulative dose intrathecal methotrexate and/or cytarabine (mL), and cranial radiation dose (per 10 Gy).

§ *P* values were calculated using general linear models; all tests were two-sided.

**Table 4.** Associations between insomnia and neurocognitive performance with adjustment for chronic health conditions, lifestyle factors, and emotional distress

Outcome variable	Referent = no sleep disturbance, no daytime fatigue or sleepiness	Female*		Male†	
		β‡	*P*	β‡	*P* §
Verbal reasoning	Daytime sleepiness and/or fatigue	0.12	.52	−0.14	.43
	Sleep disturbance	−0.18	.20	−0.23	.08
	Insomnia	−0.46	.003	−0.05	.78
Verbal learning	Daytime sleepiness and/or fatigue	0.10	.60	0.28	.19
	Sleep disturbance	−0.07	.61	0.01	.92
	Insomnia	−0.19	.26	−0.30	.16
Short-term verbal memory	Daytime sleepiness and/or fatigue	0.21	.30	0.25	.20
	Sleep disturbance	−0.14	.35	−0.03	.85
	Insomnia	−0.01	.97	−0.27	.17
Long-term verbal memory	Daytime sleepiness and/or fatigue	0.28	.20	0.37	.06
	Sleep disturbance	−0.06	.73	0.03	.85
	Insomnia	−0.13	.50	−0.21	.30
Memory span	Daytime sleepiness and/or fatigue	0.08	.67	0.13	.50
	Sleep disturbance	−0.17	.21	0.03	.83
	Insomnia	−0.20	.20	−0.22	.24
Focused attention	Daytime sleepiness and/or fatigue	0.10	.53	−0.02	.93
	Sleep disturbance	0.15	.19	−0.03	.81
	Insomnia	−0.04	.76	−0.12	.52
Sustained attention	Daytime sleepiness and/or fatigue	−0.07	.77	0.47	.04
	Sleep disturbance	−0.10	.52	0.05	.76
	Insomnia	−0.57	.002	−0.48	.04
Inattention	Daytime sleepiness and/or fatigue	−0.08	.71	0.28	.22
	Sleep disturbance	−0.10	.55	−0.10	.56
	Insomnia	−0.44	.01	−0.52	.03
Selective attention	Daytime sleepiness and/or fatigue	0.17	.39	0.22	.19
	Sleep disturbance	−0.03	.83	−0.13	.27
	Insomnia	0.01	.97	−0.26	.12
Cognitive flexibility	Daytime sleepiness and/or fatigue	0.23	.31	0.46	.06
	Sleep disturbance	0.31	.07	0.05	.75
	Insomnia	−0.17	.38	−0.34	.17
Cognitive fluency	Daytime sleepiness and/or fatigue	−0.05	.79	−0.09	.66
	Sleep disturbance	0.21	.12	−0.15	.29
	Insomnia	−0.11	.49	−0.26	.20
Working memory	Daytime sleepiness and/or fatigue	0.14	.34	0.10	.55
	Sleep disturbance	−0.00	.99	−0.27	.02
	Insomnia	−0.24	.07	−0.15	.38
Visuomotor processing speed	Daytime sleepiness and/or fatigue	0.05	.75	0.14	.45
	Sleep disturbance	0.09	.50	−0.01	.93
	Insomnia	−0.29	.05	−0.07	.71
Cognitive processing speed	Daytime sleepiness and/or fatigue	0.19	.26	0.18	.34
	Sleep disturbance	0.19	.15	−0.08	.53
	Insomnia	−0.19	.20	−0.15	.43
Fine motor speed	Daytime sleepiness and/or fatigue	0.15	.41	0.21	.30
	Sleep disturbance	0.07	.61	−0.02	.87
	Insomnia	−0.10	.52	−0.25	.25

* Female models are based on a sample of n = 435 survivors with complete sleep and neurocognitive data; additional missing data due to covariates n = 125.

† Male models are based on a sample of n = 398 survivors with complete sleep and neurocognitive data; additional missing data due to covariates n = 111.

‡ Standardized β of neurocognitive measures are based on Z scores (Mean = 0, [SD = 1]). Separate models for each neurocognitive outcome, adjusted for age at survey (years), anxiety (yes/no), depression (yes/no) pain (yes/no), physical inactivity (yes/no), and moderate to severe cardiac, endocrine, and pulmonary conditions (yes/no).

^§^ *P* values were calculated using general linear models; all tests were two-sided.

## Discussion

To our knowledge, this is the first study to examine associations between insomnia and performance-based neurocognitive outcomes in a large, heterogeneous cohort of adult survivors of childhood cancer. Moreover, the detailed phenotyping of the cohort allowed for evaluation of the impact of insomnia independent of cancer diagnosis and treatment factors. Our results support that sleep disturbances are a prevalent late effect of childhood cancer and indicate that insomnia (poor sleep efficiency combined with daytime impairment) has a substantial and detrimental impact on neurocognitive performance. Insomnia, therefore, is a potential behaviorally modifiable intervention target to improve neurocognitive performance in adult survivors of childhood cancer.

In our sample, 59% of survivors reported a sleep complaint defined as either poor sleep efficiency and/or daytime fatigue or sleepiness. Moreover, 17% of our sample met criteria for insomnia by reporting a poor sleep efficiency with daytime fatigue or sleepiness. Although comparing insomnia prevalence across studies is difficult due to inconsistency in definitions, previous studies estimate a 10–30% population-based prevalence of insomnia and a prevalence of 9–15% when considering insomnia with daytime impairment (44,45). In one study of adult survivors of non-central nervous system tumors, the prevalence of insomnia, defined as self-reported sleep efficiency less than 85%, was 28% (10). Considering both population-based estimates (44,45) and previous research in adult survivors of childhood cancer (10), our findings support that survivors experience an increased prevalence of sleep disturbance when defined by poor sleep efficiency (45%), even more substantial than previously suggested.

Given the heightened prevalence of sleep disturbances among adult survivors of childhood cancer, it is important to understand potential functional consequences and to examine what components of the sleep disturbance (ie, daytime impairment, poor sleep efficiency, or the combination) drive such functional consequences. Our cross-sectional assessment determined that insomnia was associated with poorer neurocognitive performance, whereas the presence of a sleep disturbance without daytime fatigue or sleepiness and daytime fatigue or sleepiness alone were not consistently associated with decreased neurocognitive performance. These associations were observed even after adjustment for cranial radiation therapy, neurotoxic chemotherapies, chronic health conditions, lifestyle factors, and mood disturbances known to contribute to cognitive functioning. For example, survivors with insomnia performed approximately one-third to one full SD worse on measures of attention and verbal memory. Importantly, neurocognitive deficits in these domains have been associated with a higher likelihood of survivors not graduating from college, being unemployed, and not living independently (5,6).

Although past research on sleep-related neurocognitive impairment has yielded mixed results (11), when statistically significant associations are identified they have primarily been reported in the domains of attention and memory (46). The mechanisms underlying the association between insomnia and neurocognitive impairment have not been fully elucidated, though neuroendocrine and inflammatory processes have been implicated. Individuals suffering from insomnia have been shown to have increased levels of daytime and nighttime cortisol as well as increased levels of interleukin-6, which have been linked to neurocognitive impairment (47,48). Our findings suggest a complex relationship between insomnia and neurocognition, because statistically significant associations between insomnia and domains of neurocognitive performance varied by sex and according to covariate adjustment (ie, psychological distress, neurotoxic treatment exposure, and chronic health conditions). It is unclear whether sleep disturbances globally affect all neurocognitive domains or whether some aspects of neurocognition may be more vulnerable to sleep disruptions than others (49). Likely there are several direct and/or indirect pathways through which insomnia may impair neurocognition, and the presence of other physiological and psychological factors may temper these associations. Moreover, we identified sex differences in insomnia and neurocognitive performance. This is consistent with findings in the general population, with females experiencing higher rates of insomnia (41). With respect to neurocognitive function, research suggests that females perform better on tasks of episodic memory and processing speed and males perform better on measures of spatial ability. Unfortunately, our study did not include specific measures of episodic memory or spatial ability; however, consistent with the general population, females did perform better than males on measures of processing speed (50).

Although the relationship between sleep and neurocognitive functioning is not fully understood, sleep is a health behavior amenable to intervention. Several interventions, such as cognitive-behavioral therapy for insomnia (CBT-I) (51,52), adaptations of CBT-I specific to cancer survivors (53,54), tai chi (55), and yoga (56), have all demonstrated efficacy in improving sleep disturbances and/or daytime dysfunction among samples of cancer survivors. Moreover, insomnia interventions, namely CBT-I, have been associated with improvements in anxiety and depression in both the general population (57,58) and among cancer survivors (59–61). Thus, treating insomnia may lead to improvements in other aspects of health as well as improve neurocognitive function. Future research should examine if improving sleep remediates neurocognitive late effects and intervention strategies that best achieve these gains.

The results of our study should be considered in the context of several limitations. Despite the strength of recruiting a large sample, survivors were from a single institution and were recruited for an intervention targeting sleep and cognition, which may limit the generalizability of our findings. Additionally, the cross-sectional design of the study does not allow us to infer causality. Although sleep deprivation research supports the causal direction of disrupted sleep impairing neurocognition (49), less is known about the direction of associations between insomnia and neurocognition in adult survivors of childhood cancer, who are vulnerable to both sleep and neurocognitive late effects. Beyond insomnia, there are other dimensions of sleep, such as short sleep duration, or other diagnosed sleep disorders (eg, sleep apnea or hypersomnia) that may also be associated with neurocognitive performance. It is possible that self-reported daytime fatigue and/or sleepiness in our sample serves as a marker for other sleep disorders. However, assessment of such would require the use of polysomnography and/or a multiple sleep latency test to be able to fully ascertain the range of sleep disorders in our sample. These limitations notwithstanding, our data provide strong evidence of the association between insomnia and neurocognitive performance in adult survivors of childhood cancer and support the need for further research in this area.

Insomnia is highly prevalent and contributes to the neurocognitive burden experienced by survivors of childhood cancer. Insomnia should be assessed and treated throughout the course of survivorship. Established treatments for insomnia, including cognitive behavioral therapy, are readily available and can be accessed and delivered in person or remotely (54,62). Given the observed link between insomnia and memory, processing speed, and attention problems, successful treatment of insomnia may improve neurocognitive functioning in adult survivors of childhood cancer.

## Funding

This work was supported by the National Cancer Institute at the National Institutes of Health (CA195547, M. Hudson and L. Robison, Principal Investigators; CA239689, T. Brinkman and K. Krull, Principal Investigators) and by the National Cancer Institute Training in Pediatric Cancer Care Survivorship award (5T32CA225590, K. Krull, Principal Investigator). Support to St. Jude Children’s Research Hospital was also provided by the Cancer Center Support (CORE) grant (CA21765, C. Roberts, Principal Investigator) and the American Lebanese Syrian Associated Charities (ALSAC).

## Note

No funding source was involved in the design of the study; analysis, and interpretation of the data; the writing of the manuscript; and the decision to submit the manuscript for publication.

Disclosures: The authors have no conflicts of interest to disclose.

## Supplementary Material

pkaa008_Supplementary_Data
