# Supplementary material for: Insomnia and Neurocognitive Functioning in Adult Survivors of Childhood Cancer
Source: JNCI Cancer Spectr. 2020 Feb 19;4(3):pkaa008. doi: 10.1093/jncics/pkaa008 (PMC7197383; doi:10.1093/jncics/pkaa008)
Supplement: pkaa008_Supplementary_Data [file pkaa008_supplementary_data.pdf]

Supplementary File

Supplementary Table 1. Intervention study inclusion and exclusion criteria

Inclusion Criteria	Exclusion Criteria[†]
1) Previously treated at SJCRH and a participant in SJLIFE*	1) Known allergy to melatonin or any ingredients of the study product or placebo
2) 10 or more years from diagnosis	2) Currently taking Melatonin
3) FSIQ score > 79	3) Known sleep apnea
4) ≥ 18 years of age	4) Known medically treated sleep disorder
5) Able to speak and understand the English language	5) Known diabetes mellitus – insulin treated
	6) Uncontrolled seizure disorder in the past 12 months
	7) Reported current illicit drug or alcohol abuse or dependence
	8) Reported current major psychiatric illness (i.e. schizophrenia, bipolar disorder)
	9) Current treatment with:<br>a. Benzodiazepines or other CNS depressants<br>b. Fluvoxamine<br>c. Anticoagulants (e.g. Coumadin)<br>d. Immunosuppressant or corticosteroids<br>e. Nifedipine
	10) Employed in a position that requires night work (i.e. 10pm to 6am)
	11) Females who are pregnant or lactating/nursing
	12) History of neurologic event unrelated to cancer or its treatment
	13) Sensory impairment (vision, hearing) that prohibits completion of neurocognitive examination

*SJCRH = St. Jude Children's Research Hospital; SJLIFE = St. Jude Lifetime Cohort Study
[†]Exclusion criteria for the study were informed by the subsequent intervention that followed the completion of baseline testing.

Supplementary Table 2. Prevalence of neurocognitive impairment* by sex

Neurocognitive Domain	Total Sample n=906 n (%)	Female n=470 n (%)	Male n=436 n (%)
Verbal Reasoning	139 (15.3)	80 (17.0)	59 (13.5)
Verbal Learning	106 (11.7)	62 (13.2)	44 (10.1)
Short Term Verbal Memory	99 (11.0)	58 (12.4)	41 (9.4)
Long Term Verbal Memory	133 (14.7)	73 (15.6)	60 (13.8)
Memory Span	60 (6.6)	37 (7.9)	23 (5.3)
Focused Attention	69 (7.6)	27 (5.7)	42 (9.7)
Sustained Attention	156 (17.4)	87 (18.6)	69 (16.0)
Inattention	120 (13.4)	69 (14.8)	51 (11.8)
Selective Attention	67 (7.5)	59 (12.6)	8 (1.9)
Cognitive Flexibility	163 (18.0)	72 (15.3)	91 (21.0)
Cognitive Fluency	145 (16.0)	67 (14.3)	78 (17.9)
Working Memory	32 (3.5)	15 (3.2)	17 (3.9)
Visuomotor Processing Speed	120 (13.3)	41 (8.8)	79 (18.2)
Cognitive Processing Speed	69 (7.7)	30 (6.4)	39 (9.1)
Fine Motor Speed	174 (19.3)	66 (14.1)	108 (24.9)

*Impairment defined as a z-score < -1.28.

Supplementary Table 3. Associations between insomnia and neurocognitive performance with adjustment for chronic health conditions and lifestyle factors (without anxiety and depression)

Outcome variable	Referent= No Sleep Disturbance, No Daytime Fatigue/Sleepiness	Female		Male	
		β*	*P*-value	β*	*P*-value
Verbal Reasoning	Daytime Sleepiness and/or Fatigue	0.10	0.63	-0.03	0.85
	Sleep Disturbance	-0.22	0.09	-0.23	0.06
	Insomnia	-0.60	<0.001	-0.13	0.45
Verbal learning	Daytime Sleepiness and/or Fatigue	0.13	0.44	0.20	0.29
	Sleep Disturbance	-0.11	0.45	-0.01	0.95
	Insomnia	-0.32	0.04†	-0.34	0.08
Short-term verbal memory	Daytime Sleepiness and/or Fatigue	0.18	0.87	0.13	0.44
	Sleep Disturbance	-0.21	0.15	-0.01	0.97
	Insomnia	-0.16	0.30	-0.34	0.06
Long-term verbal memory	Daytime Sleepiness and/or Fatigue	0.25	0.20	0.22	0.21
	Sleep Disturbance	-0.11	0.47	0.04	0.77
	Insomnia	-0.26	0.12	-0.31	0.09
Memory span	Daytime Sleepiness and/or Fatigue	0.07	0.67	0.09	0.59
	Sleep Disturbance	-0.24	0.07	0.04	0.72
	Insomnia	-0.33	0.02†	-0.19	0.28
Focused attention	Daytime Sleepiness and/or Fatigue	-0.00	0.99	0.00	0.99
	Sleep Disturbance	0.04	0.71	0.02	0.86
	Insomnia	-0.19	0.13	-0.31	0.10
Sustained attention	Daytime Sleepiness and/or Fatigue	-0.08	0.69	0.32	0.10
	Sleep Disturbance	-0.14	0.35	0.07	0.62
	Insomnia	-0.54	0.001	-0.67	0.001
Inattention	Daytime Sleepiness and/or Fatigue	-0.16	0.38	0.13	0.51
	Sleep Disturbance	-0.08	0.58	-0.08	0.59
	Insomnia	-0.43	0.008	-0.78	<0.001
Selective attention	Daytime Sleepiness and/or Fatigue	0.17	0.33	0.27	0.08
	Sleep Disturbance	0.02	0.86	-0.10	0.37
	Insomnia	-0.01	0.94	-0.24	0.14
Cognitive flexibility	Daytime Sleepiness and/or Fatigue	-0.03	0.89	0.35	0.13
	Sleep Disturbance	0.18	0.28	0.15	0.37
	Insomnia	-0.45	0.02†	-0.37	0.13
Cognitive fluency	Daytime Sleepiness and/or Fatigue	0.07	0.69	-0.04	0.81
	Sleep Disturbance	0.16	0.23	-0.13	0.33
	Insomnia	-0.23	0.13	-0.22	0.24
Working memory	Daytime Sleepiness and/or Fatigue	-0.01	0.96	0.14	0.34

Outcome variable	Referent= No Sleep Disturbance, No Daytime Fatigue/Sleepiness	Female		Male	
		β*	*P*-value	β*	*P*-value
	Sleep Disturbance	-0.08	0.48	-0.24	0.03
	Insomnia	-0.32	0.007†	-0.15	0.34
Visuomotor processing speed	Daytime Sleepiness and/or Fatigue	-0.05	0.72	0.15	0.36
	Sleep Disturbance	0.05	0.69	0.02	0.86
	Insomnia	-0.41	0.002	-0.20	0.25
Cognitive processing speed	Daytime Sleepiness and/or Fatigue	0.05	0.73	0.25	0.16
	Sleep Disturbance	0.18	0.16	-0.05	0.73
	Insomnia	-0.30	0.03†	-0.21	0.25
Fine motor speed	Daytime Sleepiness and/or Fatigue	0.02	0.93	0.22	0.24
	Sleep Disturbance	-0.05	0.72	-0.05	0.72
	Insomnia	-0.20	0.17	-0.39	0.06

*Standardized βetas of neurocognitive measures are based on z-scores (M=0, SD=1). Separate models for each neurocognitive outcome, adjusted for age at survey (years), pain (yes/no), physical inactivity (yes/no), and moderate to severe cardiac, endocrine and pulmonary conditions (yes/no).

†Indicates a change in the statistical significance of the p-value compared to the models presented in Table 4.
